# Application of small extracellular vesicles in the diagnosis and prognosis of nasopharyngeal carcinoma

**DOI:** 10.3389/fcell.2023.1100941

**Published:** 2023-03-10

**Authors:** Jiali Zhang, Defa Huang, Xianbin Lan, Dongming Deng, Jijing Li, Dongzhi Zhang, Yue Li, Tianyu Zhong, Shaoping Peng

**Affiliations:** ^1^ The First School of Clinical Medicine, Gannan Medical University, Ganzhou, China; ^2^ Department of Otolaryngology, First Affiliated Hospital of Gannan Medical University, Ganzhou, China; ^3^ Laboratory Medicine, First Affiliated Hospital of Gannan Medical University, Ganzhou, China; ^4^ Precision Medicine Center, First Affiliated Hospital of Gannan Medical University, Ganzhou, China

**Keywords:** nasopharyngeal carcinoma, small extracellular vesicles, prognosis, diagnosis, biomarker

## Abstract

Nasopharyngeal carcinoma (NPC) is a malignant tumor originating from the epithelium of the nasopharynx. The disease is insidious, and most patients are diagnosed at the advanced stage, resulting in poor prognosis. Early diagnosis is important to reduce NPC mortality. Small extracellular vesicles (sEVs) are rich in a variety of bioactive molecules, such as proteins, nucleic acids, and lipids, which can participate in the physiological and pathological regulation of the body by affecting the function of target cells. Numerous studies have shown that some RNAs and proteins in sEVs of tumor origin have a key role in the development of NPC and are potential candidates for malignancy detection. Studying the relationship between the cargoes of these sEVs and NPC may help in the diagnosis of the disease. Here in this review, we summarize the application of sEVs as biomarkers in the diagnosis of NPC and their role in NPC metastasis and prognosis. In addition, we discuss possible future applications and limitations of sEVs as biomarkers.

## Introduction

Nasopharyngeal carcinoma (NPC) is a malignancy with strong ethnic and regional characteristics. It is very common in Southeast Asia, East Asia, and North Africa. It occurs at a peak age of > 45 years and is two to three times more common in men than in women (Xu et al., 2015; Chen Y. P. et al., 2019; Zhu et al., 2020). The World Health Organization has classified NPC into the following three subtypes based on histology: keratinizing squamous cell carcinoma, non-keratinizing carcinoma (either differentiated or undifferentiated), and basaloid carcinoma (Chua et al., 2016; Tang et al., 2021). Keratinizing squamous cell carcinoma is associated with Epstein–Barr virus (EBV) infection in approximately 70%–80% of the cases. Almost all cases of non-keratinizing carcinoma (either differentiated or undifferentiated) are related to EBV and occur in areas where EBV is endemic (Sinha and Gajra, 2022). Research suggests that differences in diet, lifestyle, and exposure to harmful environmental factors may be the root cause of geographic differences in the incidence of NPC (Chang et al., 2017; Lee et al., 2019; Xu et al., 2019). In 2020, approximately 133,000 people were diagnosed with NPC, and there were approximately 80,000 NPC-related deaths (a mortality rate of approximately 60%) (Sung et al., 2021). Due to the lack of effective early diagnostic indicators, most patients are diagnosed at an advanced stage and are prone to distant metastasis (Guinot et al., 2020; Brody-Camp et al., 2021). Therefore, early and accurate diagnosis is of great importance for the prevention and treatment of NPC.

Currently, screening for NPC is based on serum EBV antibody assays, plasma EBV DNA tests, imaging, and tissue biopsies (Lam et al., 2018; Mao et al., 2021; Tang et al., 2021). However, these methods have some limitations in diagnosing patients with early NPC. Serum EBV antibody tests are only effective in EBV-positive NPC patients, and there is no internationally accepted standardized procedure for plasma EBV DNA testing. Moreover, the origin of EBV DNA in the blood is not known. Imaging is only available for patients with no contraindications to imaging. Tissue biopsies are invasive, and their accuracy depends on the skill and experience of the operator (Chua et al., 2016; Chan et al., 2017; Lam and Chan, 2018; Sun et al., 2019). Therefore, non-invasive and more effective early diagnostic biomarkers for NPC are urgently needed.

Extracellular vesicle (EV) as the generic term for particles naturally released from the cell. EVs are classified into small EVs (sEVs, diameter: <200 nm), medium/large EVs (m/lEVs, diameter: >200 nm) according to their size. sEVs formed by the fusion of multivesicular bodies (MVBs) with the plasma membrane can be actively secreted by healthy, malignant, and virus-infected cells (Théry et al., 2018; van Niel et al., 2018; Xiao et al., 2019). All cells, including blood cells, immune cells, cancer cells, and stem cells, can release sEVs into various body fluids, including blood, urine, breast milk, ascitic fluid, amniotic fluid, saliva, and cerebrospinal fluid (Raposo and Stoorvogel, 2013; Teow et al., 2017; Xunian and Kalluri, 2020). sEVs are rich in proteins, nucleic acids, lipids, and other bioactive substances, and these active molecules reshape the tumor microenvironment and participate in the development of NPC (Juan and Fürthauer, 2018; Liu et al., 2021; Luo and Yi, 2021). Tumor-derived sEVs contain a variety of RNAs, such as microRNA (miRNA), long non-coding RNA (lncRNA), and circular RNA (circRNA) (Valadi et al., 2007). These RNAs hold great promise for diagnostic monitoring, prognostic assessment, and immunotherapy of NPC (Duan et al., 2019; Jiang et al., 2021; Liu et al., 2021).

sEVs are rich in proteins, including a wide range of transmembrane proteins, lipid-anchored membrane proteins, peripherally associated membrane proteins, and soluble proteins. Some proteins may have important roles in the early diagnosis, prognosis, and treatment of NPC (Pegtel and Gould, 2019; Hu et al., 2022). Studies have shown that the EBV-encoded latent membrane protein 1 (LMP1) is expressed in NPC cells and enhances the radioresistance of NPC cells possibly by affecting the infected host and modulating the tumor microenvironment (Zhang et al., 2019). Moreover, LMP1 increase the expression of syndecan-2 (SDC2) and synaptotagmin-4-like (SYTL4) through nuclear factor-κB (NF-κB) signaling and improve sEV formation and secretion (Liao et al., 2020). It has also been reported that CD63, a conserved tetraspanin protein, increases the LMP1-mediated release of sEVs (Hurwitz et al., 2017; Han et al., 2021). Increased number of sEVs result in P38 MAPK signaling activation in recipient cells, promoting the proliferation of recipient NPC cells and tumor growth (Zhang et al., 2019; Wu et al., 2020; Lo et al., 2021). Here, we summarize the relationship between sEVs and NPC and the application of sEV miRNAs and proteins in the diagnosis and prognosis of NPC.

## sEVs and EBV-associated NPC

**FIGURE 1:**
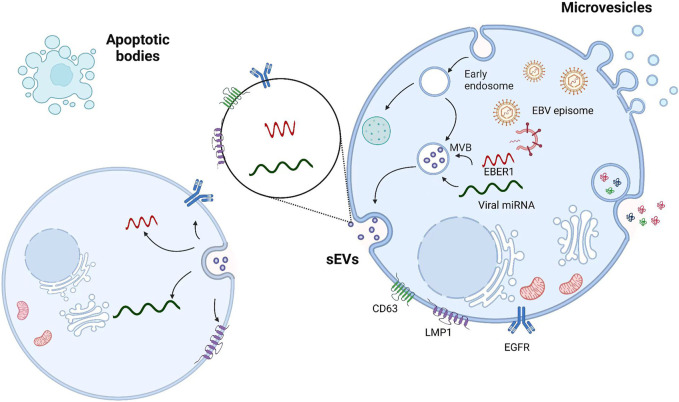
Viral particles and viral RNA are transported by sEVs from infected cells to uninfected cells. sEVs are formed by the fusion of MVBs with cell membranes; microvesicles are formed by the direct outgrowth of cell membranes; and apoptotic vesicles are formed by the shrinkage and fragmentation of apoptotic cells. The interaction between LMP1 and the tetraspanin CD63 may contribute to the selective incorporation of LMP1 into sEVs.

sEVs are generated within a subpopulation of MVBs. When sEVs are mature, MVBs are transported to the plasma membrane through microtubules, docking, and membrane fusion, resulting in sEV release into the extracellular space. sEVs influence cells in the microenvironment by interacting with the extracellular matrix. sEVs can also enter the circulation *via* lymph or blood (Bebelman et al., 2018; Théry et al., 2018; Verweij et al., 2018). In the tumor microenvironment, sEVs are key mediators of intercellular communication, regulating the pathophysiological processes of tumors (Zhang and Yu, 2019; Shehzad et al., 2021). NPC-derived sEVs significantly induce macrophages to produce the inflammatory cytokine interleukin-6 (IL-6), which activates signal transducer and activator of transcription 3 (STAT3) to promote malignant behavior of NPC cells (Han et al., 2021; Wang et al., 2021; Chen et al., 2022), such as immune escape, angiogenesis, and metastasis (Vader et al., 2014). Similarly, viruses can be transmitted through sEVs. In EBV-infected patients, EBV particles are transmitted between B cells and epithelial cells through sEVs, and specific RNAs and proteins are incorporated in sEVs to regulate intercellular signaling (Meckes et al., 2010; Raab-Traub and Dittmer, 2017; Zhao et al., 2019) (Figure 1). EBV-encoded LMP1 promotes NPC cell metastasis through store-operated calcium entry (SOCE) conduction of cytoplasmic calcium ions (Wei et al., 2015; Wei et al., 2020).

Many miRNAs in tumor-derived sEVs can promote angiogenesis in NPC through different pathways. For example, Studies have shown that miR-9 in sEVs is strongly associated with NPC patients’ prognosis and survival. miR-9 can directly inhibit the expression of its target gene *midkine* (*MDK*) in endothelial cells, and it can inhibit endothelial angiogenesis by regulating the PDK/AKT signaling pathway (Lu et al., 2018; Ramayanti et al., 2019; Wang et al., 2020a). miR-17-5p promotes neoangiogenesis in NPC by downregulating bone morphogenetic protein and activin membrane-bound inhibitor (BAMBI) and regulating AKT/vascular endothelial growth factor A (VEGF-A) signaling (Duan et al., 2019). miR-23a plays an important role in mediating angiogenesis by targeting testis-specific gene antigen 10 (TSGA10) (Bao et al., 2018). In addition, limb-bud and heart (LBH) of sEVs inhibits epithelial–mesenchymal transition (EMT) progression and angiogenesis in the NPC microenvironment. This is mainly achieved by regulating VEGF-A expression and secretion and associated signaling (Bebelman et al., 2018; Luo and Yi, 2021; Wu et al., 2022). sEV-derived EBV-encoded small RNAs (EBERs) regulate vascular cell adhesion molecule 1 (VCAM-1) expression *via* TLR3/RIG-I to induce angiogenesis (Ahmed et al., 2014; Cheng et al., 2019; Li et al., 2019). Hematopoietic cell-specific substrate protein 1-associated protein X-1 (HAX1) and phosphofructo-2-kinase/fructose-2,6-bisphosphatase 3 (PFKFB3), which are abundant in sEVs secreted by NPC cells, promote angiogenesis and accelerate the growth of NPC *in vitro* and *in vivo* (You et al., 2016; Gu et al., 2017). Thus, sEVs are closely associated with EBV transmission in humans, promoting the development of EBV-associated NPC and influencing the metastasis and prognosis of NPC.

## sEVs as diagnostic biomarkers for NPC

**FIGURE 2:**
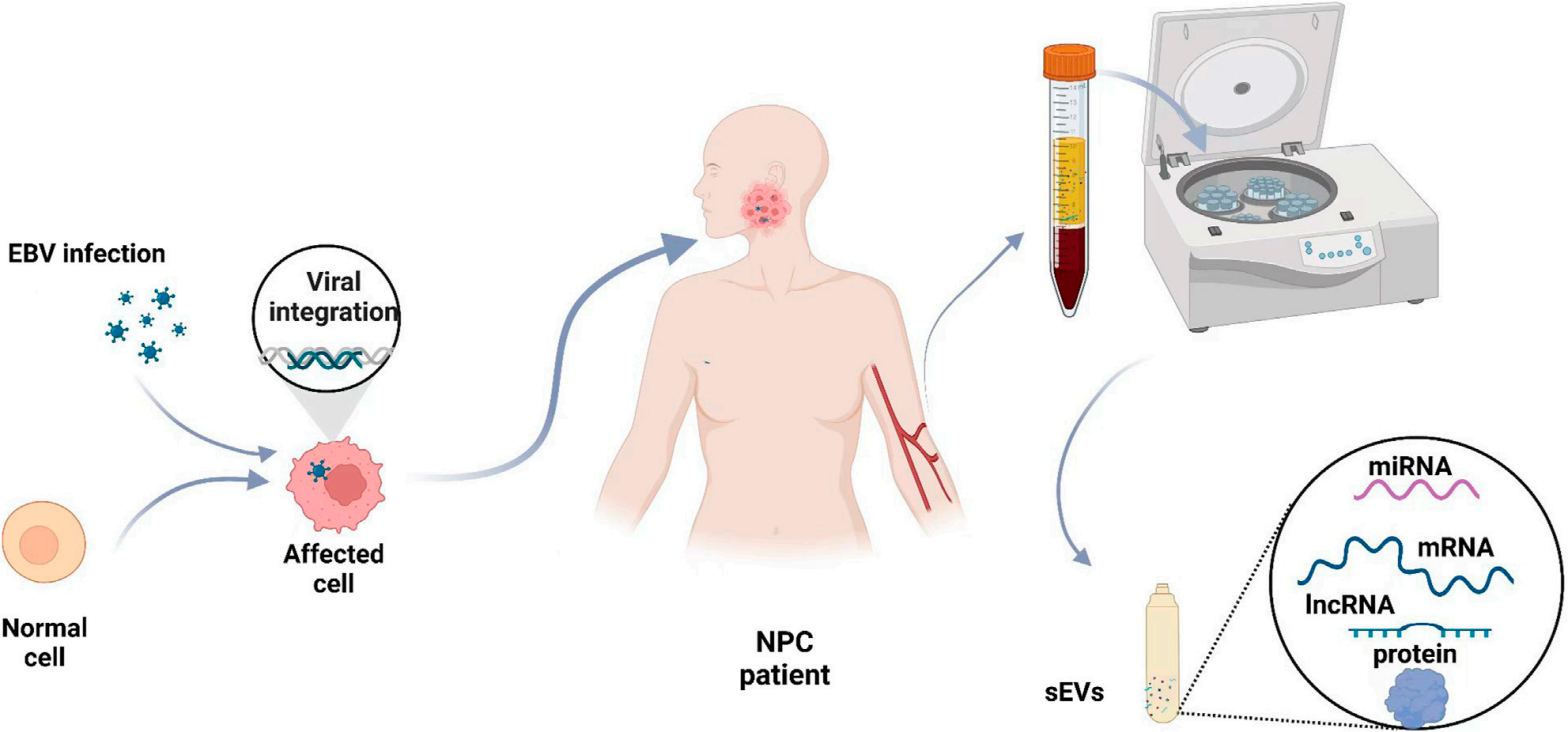
Blood small extracellular vesicles as diagnostic biomarkers for NPC. The levels of some sEV cargoes, such as miRNAs, mRNAs, lncRNAs, and proteins, are abnormal in NPC patients. Blood samples are taken from NPC patients, and sEVs are isolated to analyze the levels of various molecules.

During the latent phase of NPC infection, EBV releases various products, including Epstein–Barr nuclear antigen 1 (EBNA1), LMP1, lncRNAs (BARTs), small RNAs (EBERs), and miRNAs (EBV-miR-BART) (Tsao et al., 2017; Lo et al., 2021). Many studies have shown that miRNA, RNA, and proteins from sEVs of different origins have important roles in patients with NPC and that these molecules may serve as diagnostic indicators (Rahbarghazi et al., 2019; Liao et al., 2021; Lo et al., 2021) (Figure 2).

## sEV miRNAs

miRNAs are a small, highly conserved class of non-coding RNAs that can be used to diagnose NPC with high accuracy and specificity (Zhang and Lu, 2019; Kang et al., 2021; Liao et al., 2021). Studies have shown that sEVs of EBV BART-microRNAs secreted by NPC cells can spread from tumor sites into the peripheral blood. sEVs are sufficiently stable in blood and can transmit information through miRNAs, which may serve as novel tumor biomarkers (Wang et al., 2017; Di Santo et al., 2022; Lin et al., 2022).

Zou et al. (2020) identified five miRNAs that were significantly upregulated in EVs secreted by patients with NPC (let-7b-5p, miR-140-3p, miR-192-5p, miR-223-3p, and miR-24-3p), constituting a signature for NPC diagnosis with high sensitivity and specificity. Li et al. performed a comprehensive study of sEV-derived miRNAs and identified three miRNAs (miR-134-5p, miR-205-5p, and miR-409-3p) constituting a signature for NPC diagnosis (Jiang et al., 2021). Previous studies have shown that miR-205-5p is upregulated in NPC tissue or plasma; miR-205-5p is considered a potential diagnostic biomarker (Luan et al., 2016; Zhang et al., 2020). It was found that circulating miR-409-3p could serve as a biomarker for lung adenocarcinoma and prostate cancer (Wang et al., 2020b; Karadag et al., 2021). Jiang et al. (2021) conducted gene sequencing, selected seven candidate miRNAs, performed Gene Ontology and Kyoto Encyclopedia of Genes and Genomes pathway enrichment analyses, and constructed UpSet plots and assessment scales. Finally, they developed a model with an area under the receiver operating characteristic (ROC) curve value of 0.91, a sensitivity of 90%, and a specificity of 80%. In addition, their model can distinguish between patients with NPC at different clinical stages or with different EBV infection status and healthy people (Jiang et al., 2021). Ramayanti et al. (2019) found that BRAT13-3p, which binds to sEVs, is a promising biomarker in hematologic minimally invasive diagnostics, with an area under curve (AUC) value of 0.9 for BART13-3p miRNA in NPC patients. They identified patients with endemic and non-endemic nasopharyngeal cancer by measuring serum EV-bound BART13-3p levels, which could even be used as part of a screening strategy to diagnose NPC in endemic areas. In conclusion, these studies suggest that some miRNAs in sEVs can be used for the early diagnosis of NPC.

## sEV proteins

Many studies have shown differences in the levels of proteins in blood sEVs of NPC patients. An earlier proteomic analysis of plasma sEVs in NPC patients showed upregulation of 51 proteins and downregulation of 89 proteins (Chan et al., 2015). sEVs from EBV-infected NPC cells contain hypoxia-induced factor-1α (HIF-1α) and LMP1, which can accelerate tumor development and metastasis. LMP1 has been shown to indirectly affect the composition of sEVs (Meckes et al., 2013; Teow et al., 2017). In addition, studies have identified both LMP1 and LMP2A, which are encoded by EBV, in EBV-infected cells. A study developed a model, called EVsum5, based on the combination of LMP1, LMP2A, and the tumor markers programmed death 1 (PD-L1), epidermal growth factor receptor (EGFR), and epithelial cell adhesion molecule (EpCAM) to identify five EV subpopulations. Furthermore, the study concluded that EVsum5 performed best in NPC diagnosis, with an AUC value of 1.0, a sensitivity of 100%, and a specificity of 100% (Hu et al., 2022).

In addition, studies have shown that the *basic leucine zipper ATF-like transcription factor 2* (*BATF2*) gene is associated with a variety of malignant mechanisms. Immunohistochemistry (IHC) microarrays have been performed to determine the diagnostic value of BATF2 protein expression in NPC tissue. The results showed that BATF2 is downregulated in NPC, and the performance of serum and sEV BATF2 levels in the diagnosis of NPC was assessed using receiver operating characteristic (ROC) curve analysis. Interestingly, the sensitivity, specificity, and AUC value of plasma-derived sEV BATF2 in distinguishing between NPC patients and healthy controls were 81%, 82%, and 0.8983, respectively (Cui et al., 2021). In addition, several studies have shown that CD109 is highly expressed in both NPC cell lines and tumor tissues (Zhou et al., 2019). Applying the novel aptamer-CRISPR/Cas12a assay, Li et al. demonstrated that CD109^+^ EV and EGFR^+^ EV levels are much higher in NPC supernatant and plasma than in their normal counterparts. It is suggested that serum CD109^+^ and EGFR^+^ EVs could be used as biomarkers for nasopharyngeal cancer. A probability curve was constructed for the combination of CD109^+^ and EGFR^+^ tumor-derived EVs based on binary logistic regression (Logit (<b12>p = NPC) = −13.348 + 0.002 CD109 + 0.004 EGFR), with an AUC of 0.934, a sensitivity of 84.1%, and a specificity of 85% for NPC versus healthy subjects (Jia et al., 2016; Sakakura et al., 2016; Sunagawa et al., 2016; Mii et al., 2019; Li H. et al., 2021). Circulating EV procyclin A (CYPA), with peptidyl prolyl cis-trans isomerase (PPIase) activity, is involved in protein folding and transport, binds to membrane receptors or intracellular membrane chaperones, and activates downstream signaling pathways (Saleh et al., 2016). sEVs have much higher CYPA levels than plasma and are novel and promising biomarkers for NPC, with an AUC value of 0.844; combining CYPA protein analysis with EBV-VCA-IgA antibody assays can significantly improve the diagnosis of nasopharyngeal cancer (Yang et al., 2014; Liu et al., 2019).

## Plasma sEVs in the prognosis of NPC

Previous studies have found that the occurrence and development of NPC are closely associated with differentially expressed RNAs and proteins in sEVs. These molecules can influence the occurrence, metastasis, chemoresistance, and recurrence of NPC and are expected to serve as potential diagnostic markers (Wang et al., 2017).

### Confirmation of metastasis

Metastasis is not only a major obstacle to the clinical management of NPC but also a major cause of death in patients with NPC. Early diagnosis of NPC is important to improve patient survival.

It has been shown that overexpression of miR-34c in sEVs inhibits the development of NPC by targeting β-catenin, which is involved in proliferation, migration, invasion, and EMT (Okada et al., 2019; Wan et al., 2020). Lu et al. (2018) showed that sEVs overexpressing miR-9 inhibit angiogenesis and metastasis in NPC by targeting the pro-angiogenic protein MDK and regulating the PDK/AKT signaling pathway. In addition, another study showed that the glycolytic regulator PFKFB3 is overexpressed in NPC and that sEVs associated with NPC are highly enriched in PFKFB3, which could activate the ERK/AKT pathway and affect the proliferation, migration, and apoptosis of NPC cells (Gu et al., 2017). Hypoxic NPC cells secrete sEVs containing HIF-1α-activated matrix metalloproteinase-13 (MMP-13), which enhances migration and invasiveness, induces microenvironmental changes, and promotes NPC invasiveness (You et al., 2015). Yin et al. (2021) found that HIF-1α in adipocytes promotes CNE-2 cell metastasis by inhibiting miR-433-3p expression in hypoxic adipocyte-derived EVs (Shan et al., 2018). Furthermore, it has been demonstrated that EGFR is highly expressed in tumor tissues from distant metastatic NPC patients and is associated with a reduction in reactive oxygen species (ROS). Importantly, EGFR-enriched EVs promote the metastatic potential of NPC cells by downregulating intracellular ROS levels *via* the PI3K/AKT pathway (Li et al., 2020). sEVs secreted by NPC cells are rich in HAX1, which can be an important biomarker for NPC metastasis. HAX1 is associated with lymph node metastasis, metastasis classification, clinical staging, and poor prognosis. HAX1 regulates the focal adhesion kinase (FAK) pathway, affects microvascular formation, and promotes NPC metastasis by increasing the translation efficiency of integrin β6 (ITGB6) (You et al., 2016; You et al., 2022).

### Identification of chemoradiotherapy resistance

Nasopharyngeal cancer treatment includes surgery and non-surgical treatment modalities. Non-surgical treatments include radiation therapy, radiation plus chemotherapy, and molecular targeted therapy. Since NPC is highly sensitive to radiotherapy and chemotherapy, the main treatment for patients with early and locally advanced NPC is single radiotherapy or combined chemotherapy and radiotherapy (Adelstein et al., 2017; Colevas et al., 2018; Guan et al., 2020). In most cases, early-stage NPC requires only radiation therapy (Chen L. et al., 2019). The main obstacle to radiation therapy is inherent and acquired radiation resistance of cancer cells. Chemotherapy resistance is also a major obstacle in curing patients with recurrent NPC (Guan et al., 2020; Shan et al., 2022). Cisplatin is one of the commonly used chemotherapeutic agents for NPC and many other cancers (Riddell, 2018). Expression levels of miR-106a-5p in sEVs are significantly increased in the last cycle of cisplatin-based chemotherapy, and miR-106a-5p is enriched in cisplatin-resistant cell-derived EVs and promotes cisplatin resistance in NPC cells *in vivo* by regulating the ARNT2/AKT axis (Li J. et al., 2021). Recently, it has been shown that sEV endoplasmic reticulum-resident protein 44 (ERp44), which is produced upon endoplasmic reticulum (ER) stress in NPC cells, is involved in resistance to chemotherapy with platinum drugs, suggesting that ERp44 might be a new therapeutic target (Xia et al., 2021).

CircMYC, a newly identified circRNA in circulating sEVs of NPC patients, is significantly expressed in radiotherapy-resistant cells, and knockdown of circMYC increases the radiosensitivity of cells, suggesting that circMYC overexpression contributes to radiotherapy resistance. Luo et al. evaluated its diagnostic performance and reported an AUC value of 0.945, a sensitivity of 90.24%, and a specificity of 94.51%, suggesting that sEV circMYC can be used as a biomarker to discriminate between radioresistant and radiosensitive NPC patients (Luo et al., 2020). It was found that EVs of LMP1-positive NPC cells could influence the infected host and modulate the tumor microenvironment to enhance the radioresistance of NPC cells. Transmitted LMP1 subsequently exerts its oncogenic effects by activating P38 MAPK signaling in the recipient cells, and inhibition of P38 activity effectively restores the sensitivity of NPC cells to ionizing radiation (Zhang et al., 2019). In addition, it has also been shown that tumor-derived EVs may enhance the radiosensitivity of NPC by delivering miR-142-5p to radiotherapy-resistant NPC cells to inhibit the HGF/c-Met and EGF/EGFR pathways (Zhu et al., 2022). Interestingly, it has also been found that miR-34c-5p when overexpressed in sEVs can directly target the 3′-UTR region of β-linked protein mRNA and reduce the expression level of β-linked protein, thereby improving the resistance of NPC cells to radiation therapy (Wan et al., 2020). Overall, sEVs and their contents are potential indicators to differentiate NPC patients with or without radiotherapy resistance and are expected to be new therapeutic targets.

### Prediction of recurrence

Despite the availability of effective treatment strategies for NPC, relapse is a common challenge. Therefore, accurate and timely monitoring of recurrence is crucial to prolong the survival of NPC patients. There is a growing body of evidence that RNA and protein molecules in sEVs are strongly associated with the prognosis of NPC. Studies have shown that high levels of circMYC in tumor-derived sEVs may be associated with NPC recurrence. CircMYC can interact with the tumor suppressor genes miR-20b-5p and let-7e-3p to target the *CRY2* gene, which is involved in cell proliferation and apoptosis processes such as EMT, AKT signaling, and p53 signaling in tumors (Huber et al., 2016; Rahman et al., 2019; Luo et al., 2020). CYPA levels are much higher in sEVs of NPC patients than in serum, and CYPA can be used for prognosis and monitoring of EBV-associated NPC (Liu et al., 2019). Moreover, sEV miR-24-3p mediates T cell suppression through inhibition of FGF11, is involved in tumorigenesis, and may serve as a potential prognostic biomarker for NPC (Ye et al., 2016).

## Discussion

In recent years, sEVs have become an active research topic as non-invasive diagnostic and prognostic markers for NPC. sEV-derived miRNAs and proteins have been reported to play various roles in inflammation, cellular communication, tumor proliferation, angiogenesis, and metastasis. These functions highlight their potential as biomarkers (Mao et al., 2018). Nasopharyngeal cancer, which is associated with a high mortality rate, is often at an advanced stage when diagnosed. The main goal of early screening is to reduce the rate of missed diagnosis and keep the rate of misdiagnosis within an acceptable range (Ramsey et al., 2019; Feng et al., 2020). The false-positive rate of EBV-associated antibody testing limits the effectiveness of nasopharyngeal cancer screening and may have only limited value in screening patients with early-stage disease or in predicting cancer progression in those with elevated IgA antibodies (Ji et al., 2014).

sEV RNAs and proteins hold great promise as diagnostic markers for NPC (Table 1).

**TABLE 1:** Summary of the performance of blood small extracellular vesicles in NPC diagnosis.

Name	Exp	Source	AUC	*p*-value	Other	Ref.
miR-140-3p + miR-192-5p + miR-223a-3p + miR-24-3p + let-7b-5p	**↑**	Serum	---	---	Increased in NPC tissue	Zou et al. (2020)
miR-34-5p + miR-409-3p	**↓**	Plasma	0.91	<0.05	Used for early diagnosis	Jiang et al. (2021)
+ miR-205-5p	**↑**					
BART13-3p	**↑**	Serum	---	---	Used for early diagnosis	Ramayanti et al. (2019)
LMP1 + LMP2A + PD-1 + EGFR + EpCAM	**↑**	Plasma	1.0	<0.001	Used for early diagnosis	Hu et al. (2022)
BATF2	↓	Serum	0.8983	<0.05	Decreased in NPC tissue	Cui et al. (2021)
CD109 + EGFR	↑	Serum	0.934	<0.05	Used for early diagnosis and prognosis	Li H. et al. (2021)
CYPA	↑	Serum	0.844	<0.0001	Increased in NPC tissue	Liu et al. (2019)

↑, increased; ↓, decreased; ---, unrevealed; Ref., Reference; Exp, expression.

sEV markers have an important role in metastasis, prognosis, and radiotherapy resistance. Models based on combinations of multiple miRNAs provide a direction for using sEVs as efficient and accurate tumor markers in the future. Screening for the differential expression of proteins in tumor tissues and circulating sEVs is also a promising tool. In the entire process of cancer development, the early up- or downregulation of multiple markers in sEVs in NPC has an indispensable role in the prognosis of the disease. For example, the metastasis and migration capabilities of tumor cells are used for therapeutic selection of radiotherapy modalities, identification of therapeutic targets, and prediction of the risk of tumor recurrence.

sEVs have great advantages as new tumor markers. They contain a variety of bioactive molecules, have little serum interference, are highly stable, do not degrade in the extracellular environment, can be detected in a variety of humoral environments, are non-invasive to extract, and can be developed as carriers for drug and nucleic acid delivery using their biocompatibility and permeability (Arrighetti et al., 2019; Pullan et al., 2019; Tang et al., 2020). Therefore, sEVs are extremely attractive as new biomarkers. Of course, they also have some limitations. First of all, the accurate acquisition of sEVs from samples is a necessity. Current isolation and extraction techniques still have some limitations, such as cumbersome steps, and they are time-consuming and costly. Targeting receptor cells with sEVs remains very challenging (Luo and Yi, 2021). Moreover, it is necessary to further analyze the origin of sEVs in the blood of EBV-positive and -negative NPC patients. Therefore, in the future, we hope we will be able to more accurately identify specific sEVs in patients with different NPC types.

The identification of generic biomarkers based on sEVs in patients with NPC has not yet been achieved. We think that a mixture of models based on several miRNAs or proteins is more advantageous than using only one marker since it increases accuracy. To further address this issue, comparative studies with larger sample sizes are necessary. sEVs are expected to be a source of biomarkers for NPC, and it is hoped that soon, non-invasive diagnosis and extensive screening of patients based on sEVs can be achieved. It is expected that future studies will provide evidence for the large-scale clinical application of sEVs.
